# Performance of International AIDS Vaccine Initiative African clinical research laboratories in standardised ELISpot and peripheral blood mononuclear cell processing in support of HIV vaccine clinical trials

**DOI:** 10.4102/ajlm.v10i1.1056

**Published:** 2021-02-17

**Authors:** Robert K. Langat, Bashir Farah, Jackton Indangasi, Simon Ogola, Gloria Omosa-Manyonyi, Omu Anzala, Jean Bizimana, Emmanuel Tekirya, Caroline Ngetsa, Moses Silwamba, Enoch Muyanja, Paramesh Chetty, Maureen Jangano, Nancy Hills, Jill Gilmour, Len Dally, Josephine H. Cox, Peter Hayes

**Affiliations:** 1 Kenya AIDS Vaccine Initiative, Institute of Clinical Research, University of Nairobi, Nairobi, Kenya; 2 International AIDS Vaccine Initiative (IAVI), Human Immunology Laboratory, Imperial College, London, United Kingdom; 3 Projet San Francisco, Kigali, Rwanda; 4 Kenya Medical Research Institute Centre for Geographical Medicine Research Coast, Mombasa, Kenya; 5 Zambia EMORY HIV Research Project, Lusaka, Zambia; 6 Ugandan Virus Research Institute-IAVI, Entebbe, Uganda; 7 International AIDS Vaccine Initiative, Johannesburg, South Africa; 8 Clinical Laboratory Services, Johannesburg, South Africa; 9 School of Medicine, University of California, San Francisco, California, United States; 10 Emmes Corporation, Rockville, Maryland, United States; 11 Clinical Trials Program, Vaccine Research Center, National Institutes of Health, Bethesda, Maryland, United States

**Keywords:** PBMC processing, peripheral blood mononuclear cells, ELISpot, clinical trials, good clinical laboratory practice, proficiency testing

## Abstract

**Background:**

Standardisation of procedures for performing cellular functional assays across laboratories participating in multicentre clinical trials is key for generating comparable and reliable data.

**Objective:**

This article describes the performance of accredited laboratories in Africa and Europe on testing done in support of clinical trials.

**Methods:**

For enzyme-linked immunospot assay (ELISpot) proficiency, characterised peripheral blood mononuclear cells (PBMCs) obtained from 48 HIV-negative blood donors in Johannesburg, South Africa, were sent to participating laboratories between February 2010 and February 2014. The PBMCs were tested for responses against cytomegalovirus, Epstein Barr and influenza peptide pools in a total of 1751 assays. In a separate study, a total of 1297 PBMC samples isolated from healthy HIV-negative participants in clinical trials of two prophylactic HIV vaccine candidates in Kenya, Uganda, Rwanda and Zambia were analysed for cell viability, cell yield and cell recovery from frozen PBMCs.

**Results:**

Most (99%) of the 1751 ELISpot proficiency assays had data within acceptable ranges with low responses to mock stimuli. No significant statistical difference were observed in ELISpot responses at the five laboratories actively conducting immunological analyses. Of the 1297 clinical trial PBMCs processed, 94% had cell viability above 90% and 96% had cell yield above 0.7 million per mL of blood in freshly isolated cells. All parameters were within the predefined acceptance criteria.

**Conclusion:**

We demonstrate that multiple laboratories can generate reliable, accurate and comparable data by using standardised procedures, having regular training, having regular equipment maintenance and using centrally sourced reagents.

## Introduction

Clinical trials related to HIV, malaria and tuberculosis have been conducted in Africa for many years.^1,2,3,4,5,6^ To harmonise the immunological data generated from these trials, laboratories responsible for clinical sample processing must establish standardised procedures to meet International Conference on Harmonization good clinical laboratory practice (GCLP) and the World Health Organization guidelines for collecting, processing, storing and performing of functional assays of samples such as enzyme-linked immunospot assay (ELISpot), which is used to assess the immunogenicity of vaccine candidates.

The International AIDS Vaccine Initiative (IAVI) has partnered with local institutions and established Good Clinical Laboratory Practice (GCLP)-compliant laboratories across Africa, Europe and India to conduct safety and immunogenicity assessments in support of clinical trials of HIV vaccine candidates.^7,8^ These laboratories are equipped to process and store samples for later testing and can perform ELISpot and flow cytometry immunological assays. To-date IAVI has conducted over 20 Phase 1 HIV vaccine trials (https://www.iavi.org/our-work/clinical-epidemiology-research/clinical-research-centres), most of them in Africa.^9.10,11,12^ To ensure uniformity of data, IAVI sponsored a central laboratory – the IAVI Human Immunology Laboratory (HIL) – based at Imperial College London to provide standardised operating procedures, technical training, critical assay reagents and long-term storage of samples. Additionally, IAVI and partners have built the capacity of local personnel both professionally and academically, through technical training, mentoring and funding for investigator-initiated research projects within sites. The IAVI GCLP-accredited laboratories have been used as reference laboratories by local and international research organisations for training and storage of samples on a short-term basis.

Cellular functional assays have been used for assessing the immune response to many vaccines.^13^ In this article, we focus on the interferon-gamma (IFN-*γ*) ELISpot assay and report on the results from the ELISpot proficiency scheme. IFN-*γ* ELISpot is a standard assay for measuring the immunogenicity of vaccine candidates such as for HIV and tuberculosis^13^ and has been utilised by many research groups. Although the ELISpot assay has been used in many clinical trials, it is prone to inherent variability within samples and between operators within and across laboratories.^14^ These discrepancies could be attributed to different reagents and equipment being used, inadequate training of personnel, lack of proficiency testing schemes and lack of quality management systems. Therefore, there is an urgent need to standardise this assay to generate accurate and reliable data across multiple laboratories. Previously, it was shown that multiple laboratories reported varied responses in ELISpot proficiency testing.^15^ There have been measures put in place to address this shortcoming and recent reports on ELISpot proficiency have shown incredibly improved results with concordant results between multiple GCLP-accredited laboratories.^16,17,18^ The assessment of IFN-*γ* producing T-cells in vaccinees by ELISpot is a standard measure for determining a vaccinee’s immune response to HIV-1 vaccine candidates. In the field of human T-cell immunology, IFN-*γ* has been widely used as a measure of CD4 and CD8 activation after stimulation with various peptides and IFN-*γ* is easily detected by ELISpot. Samples showing IFN-*γ* T-cell responses to test peptides are further assayed for other cytokine responses and immunological functions.

It is worth noting that most of the laboratories performing end-point IFN-*γ* ELISpot either for proficiency testing or clinical trial schemes are based either in Europe or the United States^,16,19^ which is disproportionate to where the burden of HIV/AIDS, infectious diseases and re-emerging ‘orphan’ tropical infectious diseases are predominant.^20^ There is now an increased focus on conducting vaccine trials where the pandemic is most severe; thus, networks of laboratories to support large clinical trials need to be developed in sub-Saharan Africa. On this front, IAVI with its partners pioneered the GCLP accreditation of laboratories in Africa. For external quality assurance, these GCLP-accredited laboratories were also enrolled in an ELISpot proficiency scheme. The GCLP-accredited laboratories now conduct safety and immunogenicity assays such as ELISpot and flow cytometry for the assessment of HIV vaccine candidates and are fully equipped to collect, process and store samples for later testing.

## Methods

### Ethical considerations

The study protocol was approved by the ethics committees of Kenyatta National Hospital, University of Nairobi, Kenya (reference numbers P81/3/2010 & P298/7/2011), the Uganda Virus Research Institute, Entebbe, Uganda (GC/127/10/08/31 & GC/127/11/09/12), Projet San Francisco (PSF), Kigali, Rwanda (006/RNEC/2011), Zambia EMORY HIV Research Project, University of Zambia (008-03-10), Emory University (REC-270606-013) and South African National Blood Service (IRB00041163) and was reviewed by the responsible regulatory authorities in each country.

Each participant provided written informed consent before undertaking any study procedures. At the South African National Blood Service, blood donors signed informed consent after the procedure was explained to them. Blood collected from these donors was not labelled with donor names, but blood bag identifiers. The blood bag was further labelled at clinical laboratory services (CLS) with a project number linked to the blood bag on the laboratory information management system. Each participant in the HIV-1 clinical trial study provided written informed consent before blood samples were collected. The samples were de-identified and labelled with a study number.

### Participating laboratories

Six of the seven participating laboratories were IAVI-supported laboratories: (1) Kenya AIDS Vaccine Initiative-Institute of Clinical Research (KAVI-ICR) University of Nairobi, Nairobi, Kenya, (2) IAVI Human Immunology Laboratory, Imperial College London, United Kingdom, (3) Uganda Virus Research Institute (UVRI), Entebbe, Uganda, (4) Kenya Medical Research Institute Centre for Geographical Medicine Research Coast (KEMRI-CGMRC), Kilifi, Kenya, (5) Zambia EMORY HIV Research Project (ZEHRP), Lusaka, Zambia, and (6) Projet San Francisco (PSF), Kigali, Rwanda. Clinical laboratory services, Witwatersrand University, Johannesburg, South Africa, is the only laboratory not supported by IAVI but contracted to coordinate the sourcing, testing and shipping of peripheral blood mononuclear cells (PBMCs) for ELISpot proficiency testing. Clinical laboratory services is accredited for both ISO 15189 and GCLP and followed the same operating procedures as the IAVI-sponsored laboratories. Clinical laboratory services also participated in ELISpot proficiency testing and is included in this report.

### Laboratory preparation

#### Process of establishing clinical trial laboratories under good clinical laboratory practice guidelines

Comprehensive training programmes, calibrated and maintained equipment and quality control measures were integral in establishing IAVI’s sponsored laboratories (Table 1) and are described in detail in Supplementary Document 1.

**TABLE 1:** Summary of the process of establishing a clinical trial laboratory under good clinical laboratory practice guidelines.

Good clinical laboratory practice guideline	Process
Development and qualification of collaborating laboratories	Assessment of laboratories’ needs, development of required infrastructure, transfer and qualifications of assays
Qualification and validation of equipment and assays	Develop qualification and validation plans, generate data and review compared against predefined criteria
Equipment service and maintenance	Develop calibration plans and document calibration and maintenance of all critical equipment
Development of essential documents	Develop and review standard operating procedures and other supporting documents describing safety and immunogenicity assessments
Reagent and consumable procurement	Critical reagents are purchased from approved vendors according to an approved standardised specification
External quality assurance programme	Develop quality assessment programme covering all safety testing parameters, processing, storage and shipment of PBMCs and the ELISpot assay
Training programme	Good clinical laboratory practice and technical trainings to ensure compliance with international standards for conducting clinical trials
Evaluation and accreditation	Good clinical laboratory practice compliance and acceptable technical performance monitoring by a comprehensive audit programme
On-going technology transfer	Transfer of new assays to African clinical trial laboratories and the establishment of separate research programmes.

PBMCs, peripheral blood mononuclear cells; ELISpot, enzyme-linked immunospot assay.

To minimise potential failures in the IAVI laboratories, quality control systems and operating procedures were put in place and corrective actions were instituted whenever a laboratory encountered a technical or assay failure to prevent future re-occurrence. Two laboratories (HIL and CLS) were designated to provide laboratory support and quality control management as these two laboratories were based in ideal locations to support a global clinical trial programme. Both locations are major international hubs in Europe and Africa, with direct flights to and from the IAVI-supported laboratories, thereby reducing time and cost to transport samples as well as reduce damage risk to samples while in transit.

#### ELISpot proficiency panel design

International AIDS Vaccine Initiative GCLP laboratories were enrolled in an IFN-*γ* ELISpot proficiency scheme coordinated by CLS, South Africa. Clinical laboratory services sourced buffy coat blood pack samples from the South African National Blood Service, then isolated and cryopreserved PBMCs. Frozen PBMC samples were thawed and assessed for cell viability, cell recovery and performance in ELISpot. Samples with poor viable cell recovery or performance in ELISpot were excluded. PBMC samples with 50 to 100 vials were selected to provide laboratories with identical sets of PBMC samples (six PBMC samples per set) to allow testing of the same PBMC set over 6 to 12 months (that is 7 labs over 6 months would require 42 vials of each PBMC sample; 12 months would require 84 vials). Frozen PBMC were maintained within a liquid nitrogen vapour phase freezer repository at CLS until distribution to the laboratories participating in ELISpot proficiency. Four laboratories – actively involved in clinical trials and performing cellular functional assays – conducted ELISpot monthly (KAVI-ICR, UVRI, PSF-Kigali and HIL), while three laboratories – not involved in clinical trials – conducted ELISpot quarterly (KEMRI-CGMRC, ZEHRP and CLS). PBMCs were tested against two peptide pools; (1) 32 8–10-mer peptides representing an immunodominant cluster of differentiation 8+ (CD8+) T-cell epitopes from cytomegalovirus, Epstein Barr virus and influenza virus (Anaspec Inc., California, United States),^21^ and (2) 138 15-mer peptides overlapping by 11 amino acids spanning the human cytomegalovirus pp65 protein. These peptides were chosen because the majority of the population have pre-exposed immunity against these viruses and will have detectable T-cell response to these peptides. Phytohaemagglutinin (PHA-L; Sigma L4144, Sigma, Poole, Dorset, United Kingdom) and dimethyl sulfoxide (diluted in culture medium) were used as positive and negative controls respectively. Sample processing and ELISpot assay were performed according to the minimal information about T-cell assays guidelines.^22^

#### Source of proficiency peripheral blood mononuclear cells and clinical trial samples

Peripheral blood mononuclear cells used in ELISpot proficiency testing were obtained from the buffy coat (South African National Blood Service, Johannesburg, South Africa) by Ficoll-Paque gradient centrifugation. Blood donors were screened for HIV, hepatitis B, and syphilis. Briefly, whole blood was diluted with sterile phosphate-buffered saline (PBS, Sigma-Aldrich. St. Louis, Missouri, United States) at a ratio of 1:1 and layered gently over 20 mL Ficoll density gradient (Ficoll-Hypaque PREMIUM; GE Healthcare, Uppsala, Sweden) at a ratio of 2:1 in a 50 mL Falcon tube (Greiner Bio-One, Stonehouse, United Kingdom). The tubes were centrifuged at 750 × *g* for 25 min at room temperature with the brakes off. After centrifugation, the plasma component was aspirated using a sterile Pasteur pipette (Alpha Laboratories, Eastleigh, United Kingdom) into a waste container containing bleach. The PBMC component was then removed into a new sterile 50 mL tube and washed two times with 20 mL PBS by centrifugation at 400 × *g* for 10 min with the brakes on. After washing, the cells were resuspended in 10 mL of Roswell Park Memorial Institute (RPMI) 1640 medium supplemented with 10% calf serum, 1 U/mL penicillin, 1 *μ*g/mL streptomycin, and 300 *µ*g/mL L-glutamine.

Peripheral blood mononuclear cells obtained from clinical trial participants was isolated from heparinised blood by density gradient centrifugation using Histopaque 1077 (H8889, Sigma). Briefly, 20 mL of blood was layered onto 20 mL of Histopaque in a 50 mL tube (Falcon 357522, Sarstedt, Nümbrecht, Germany) using a sterile serological pipette (Falcon, Sarstedt). The blood was then centrifuged at 400 × *g* for 40 min at room temperature with brakes off. After centrifugation, the upper plasma fraction was aspirated to within 1.5 to 2 cm of the PBMC band located at the interface between the yellow plasma fraction and the clear fluid below the PBMC band. The cells were washed in Hank’s balanced salt solution without Ca^+^ or Mg^+^ with phenol red (Sigma-Aldrich) by centrifugation at 500 × *g* for 10 min at room temperature (1st wash) with brakes on. After centrifugation, the supernatant fluids were discarded into the waste container, the cells were resuspended in Hank’s balanced salt solution and the tubes were centrifuged again at 400 × *g* for 10 min (second wash). After centrifugation, the supernatant fluids were discarded into the waste container and cells were resuspended in Hank’s balanced salt solution and centrifuged at 400 × *g* cells for 10 min at room temperature with brakes on (third wash). After centrifugation, the supernatant fluids were discarded, and cells were resuspended in Hank’s balanced salt solution for counting.

In a separate study, PBMC from clinical trial samples were obtained from heparinised blood from a healthy HIV-negative placebo and vaccine recipients participating in clinical trials of two prophylactic HIV vaccine candidates at KAVI-ICR, UVRI-IAVI, PSF and ZEHRP.^10,11^ These four laboratories participated in HIV clinical trials and the PBMC data were available for analysis. These PBMCs were processed as described above and had to meet the following predefined acceptance criteria: (1) processing of PBMC within 6 h from blood draw to cryopreservation, (2) cell viability above 90% and cell yield greater than 0.7 × 10^6^ PBMC per mL of blood for freshly isolated PBMC, (3) cell viability above 80% for frozen, thawed and overnight rested PBMC. After isolation, cells were counted using a Vi-Cell XR automated cell counter (Beckman Coulter, United Kingdom) and cryopreserved in 1 mL foetal calf serum containing 10% dimethyl sulfoxide in Nalgene system 100^TM^ cryogenic tubes (ThermoFisher Scientific, New York, United States) at a final concentration of 10–15 ×10^6^ PBMC per mL and transferred to a rate-controlled freezer (Planer, Sunbury-On-Thames, United Kingdom). This system cools the cells by a temperature decrease of 1°C per min from +4 °C to –80 °C followed by a 10 °C per min decrease until the temperature of the PBMCs was –120 °C after which the cells were transferred to vapour phase liquid nitrogen (LN) for long-term storage. PBMCs for ELISpot proficiency testing were cryopreserved in the same manner.

#### Shipment of proficiency peripheral blood mononuclear cells

Cryopreserved PBMCs were shipped to the participating laboratories as non-infectious human specimens –biological substances, category B and UN337, packed in compliance with the International Air Transport Association (IATA) packing instruction 650. Before shipping, CLS notified the receiving laboratories of the PBMC proficiency panels’ shipping itinerary so that they can be ready to appropriately store the samples upon receipt. The proficiency PBMCs were shipped on a temperature-controlled dry shipper (MVE Jencons, United Kingdom). Dry shippers were calibrated for 7 days to ensure they are in good condition for shipment of PBMCs. Briefly, on day 1, empty dry shippers were weighed and filled with LN to the brim and left overnight to adsorb. The next day, excess LN was decanted; the weight and temperature of the shipper were recorded. For the next 5 days and at the same time as the second day, the weight and temperature of the dry shipper were measured and recorded to determine the weight and temperature loss. For each dry shipper to pass calibration, its average weight and temperature loss in 24 h over the 5 days should be no more than 0.66kg (manufactureres specification is 0.6kg + 10%) and less than –190 °C. A day before shipment, the calibrated dry shippers were filled with LN and left overnight. The next day, the excess LN was decanted after which the weight and temperature of the shipper were recorded. The PBMCs were loaded onto a pre-cooled canister and placed into the shipper. The shippers were fitted with temperature loggers which were activated only after the samples were loaded to monitor the temperature of PBMCs while in transit. Once the paperwork was completed the dry shippers were collected by the IATA certified shippers (World Courier). Upon arrival at the laboratory, the loggers were removed and temperature data was downloaded and recorded. Quality checks were then done for the PBMCs against the shipment manifest that accompanied the samples and samples cryopreserved in the LN tank until use.

#### IFN-*γ* ELISpot assay

**Setup of IFN-*γ* ELISpot assay:** To minimise variations and allow investigation of any technical issue resulting from reagent use, although no such reagent issues were noted, the following measures were taken. All laboratories used the same batch of calf serum, which was pre-tested for performance in PBMC isolation, cryopreservation, cell recovery from frozen PBMC, and PHA-L and cytomegalovirus acceptable response ELISpot assay. Also, the catalogue number, lot number and expiry date of all reagents used in each assay batch were recorded, including ELISpot plates, which were obtained in batches of at least 50 plates.

**Thawing and recovery of PBMCs:** The cryopreserved PBMC panel was thawed and rested overnight in culture media. Briefly, PBMCs were removed from the LN tank and transported to the laboratory in dry ice. They were immediately immersed in a 37 °C water bath and left until a small amount of ice of the freezing media remained. The cells were aspirated into a 10 mL RPMI medium supplemented with 10% calf serum, 1 mM L-glutamine, 100 units/mL penicillin, 100 *μ*g/mL streptomycin, 1 mM sodium pyruvate and 0.5 mM HEPES (all supplements were diluted in RPMI and purchased from Sigma-Aldrich). The tube was centrifuged at 250 × *g* for 10 min room temperature, after which the cell supernatant was discarded. The cells were resuspended in 4 mL RPMI medium supplemented with 20% calf serum and plated in two wells of a 24-well plate at a concentration of 2.5 × 10^6^ PBMCs per mL. The inoculated plates were incubated overnight. The next day, cells were washed in culture media and counted.

**Plate treatment:** The 96-well filter plates (Multiscreen HTS IP MSIP4510; Millipore, United Kingdom) to be used for the assay were treated with ethanol. Briefly, 50 *µ*L of 70% ethanol was added to the wells and let to stand for 2 min. The wells were then washed three times with 200 *µ*L sterile PBS.

**Plate coating:** After the ethanol treatment, each well was coated with 100 *µ*L of 10 *µ*g/mL PBS-diluted anti-human IFN-*γ* antibody (clone 1-D1K; 1 mg/mL; Mabtech, Sweden). The plates were then incubated at 4 ^o^C overnight.

**Plate blocking:** The next day, plates were washed three times with 200 *μ*L PBS and blocked with 200 *μ*L RPMI medium supplemented with 10% calf serum per well at 37 °C for at least 2 h. After 2 h of incubation, the plates were removed and the medium was aspirated and discarded.

**Addition of antigen and inoculation of cells:** 100 *µ*L of: (1) cell medium containing 2.25 *µ*g/mL each of cytomegalovirus, Epstein-Barr virus and influenza virus (CEF) and cytomegalovirus peptide was added to the reaction; (2) mock culture (dimethyl sulfoxide + culture medium) was added to the mock wells (medium negative control); (3) cell medium containing 15 *µ*g/mL PHA-L was added to the assay positive control wells and (4) cell medium containing 2.25 *µ*g/mL cytomegalovirus peptide into the cell-free (peptide negative control) wells. PBMCs were added to all the wells except the cytomegalovirus cell-free control well at a density of 200 000 cells per 50 *µ*L culture media. Assay was done in quadruplicate. The plates were then moved to the incubator for 16 h – 24 h.

**Addition of detection antibody:** The next day, the plates were washed six times with 200 *µ*L PBS supplemented with 0.05% tween (PBS/T) using an automated washer (Bio-Tek Instruments Inc., Winooski, Vermont, United States). Detection was done by adding 100 *μ*L 1 *μ*g/mL PBS-diluted biotinylated anti-human IFN-*γ* antibody (clone 7-B6-1, 1 mg/mL) (Mabtech, Sweden) to the plates. The plates were then incubated at room temperature for 2 h. The plates were then washed six times as above after which 100 *µ*L of peroxidase avidin-biotin complex (Vector Laboratories, Burlingame, California, United States) was added to each well and incubated at room temperature for an hour. After incubation, plates were washed three times with 200 *µ*L PBS/T followed by another triple wash with 200 *µ*L PBS.

**Spot development:** 100 *µ*L of 3-amino-9-ethyl carbazole substrate solution (Vector Laboratories, Burlingame, California, United States) was added to the plates. The 3-amino-9-ethyl carbazole substrate solution was obtained by dissolving 3-amino-9-ethyl carbazole tablets in 2.5 mL DMF (VWR International, United States) after which the solution was diluted in 47 mL sterile tissue culture water supplemented with 280 *µ*L 2M acetic acid, 180 *µ*L 2M sodium acetate and 25 *µ*L hydrogen peroxide (all from Sigma-Aldrich, St. Louis, Missouri, United States). The plates were then incubated at room temperature for 4 min. The reaction was stopped by running the plates over gentle-running tap water. The underdrain of the plates was removed, and plates left to dry overnight in the dark.

**Imaging and analysis of ELISpot plates:** All of the participating laboratories in this study used the same ELISpot reader system (AutoImmun Diagnostika, Germany) with software version 4.0. It is a computer-based system for the semi-automatic interpretation of 96-well ELISpot plates. The system had the following settings for IFN-*γ*: Well(s): A1 - H12; Count settings: IFN-γ; Algorithm: v.3.2.x; Intensity MIN: 15; Size MIN: 72; and Emphasis: Small.

This plate reader system is regularly maintained by use of a master lot plate supplied alongside the reader system, which contains artificial spots designed to test the performance of the reader system. As part of the internal quality control programme, each laboratory read this control plate once a week to assess the performance of the ELISpot reader using predefined spot parameters.

The developed plates were imaged on the AID ELISpot reader system. The ELISpot data were expressed as the numbers of spot-forming cells (SFC) per million PBMC (Supplementary Document 1 Figure 2). The acceptance criteria for each assay are: (1) the average SFC in the mock wells (peptide and PHA free) must be less than 50 SFC per 10^6^ PBMC; (2) the average SFC in the negative antigen control wells (cell free) must be less than 5 SFC per well; (3) the average SFC in the assay positive control wells (peptide free + PHA) must be more than 50 SFC per 10^6^ PBMC in the positive control PHA. The plates were read and raw SFC data were submitted to HIL for evaluation by a senior scientist and results shared on the access restricted CLS website.

### Statistical analyses

This study aimed to compare the assay results obtained from the analysis of the same (frozen) PBMC samples provided to seven laboratories. We analysed the inter-lab and inter-operator variability and investigated cell recovery, viability and processing time. Outcome measures include the recovery and viability rates of frozen PBMCs, and ELISpot counts for mock, cytomegalovirus and CEF stimuli. For uniformity of data, cell recovery data after thawing and resting overnight from one clinical trial with PBMCs frozen at 15 million cells per mL/vial were normalised to 10 m cells per mL.

**FIGURE 1:**
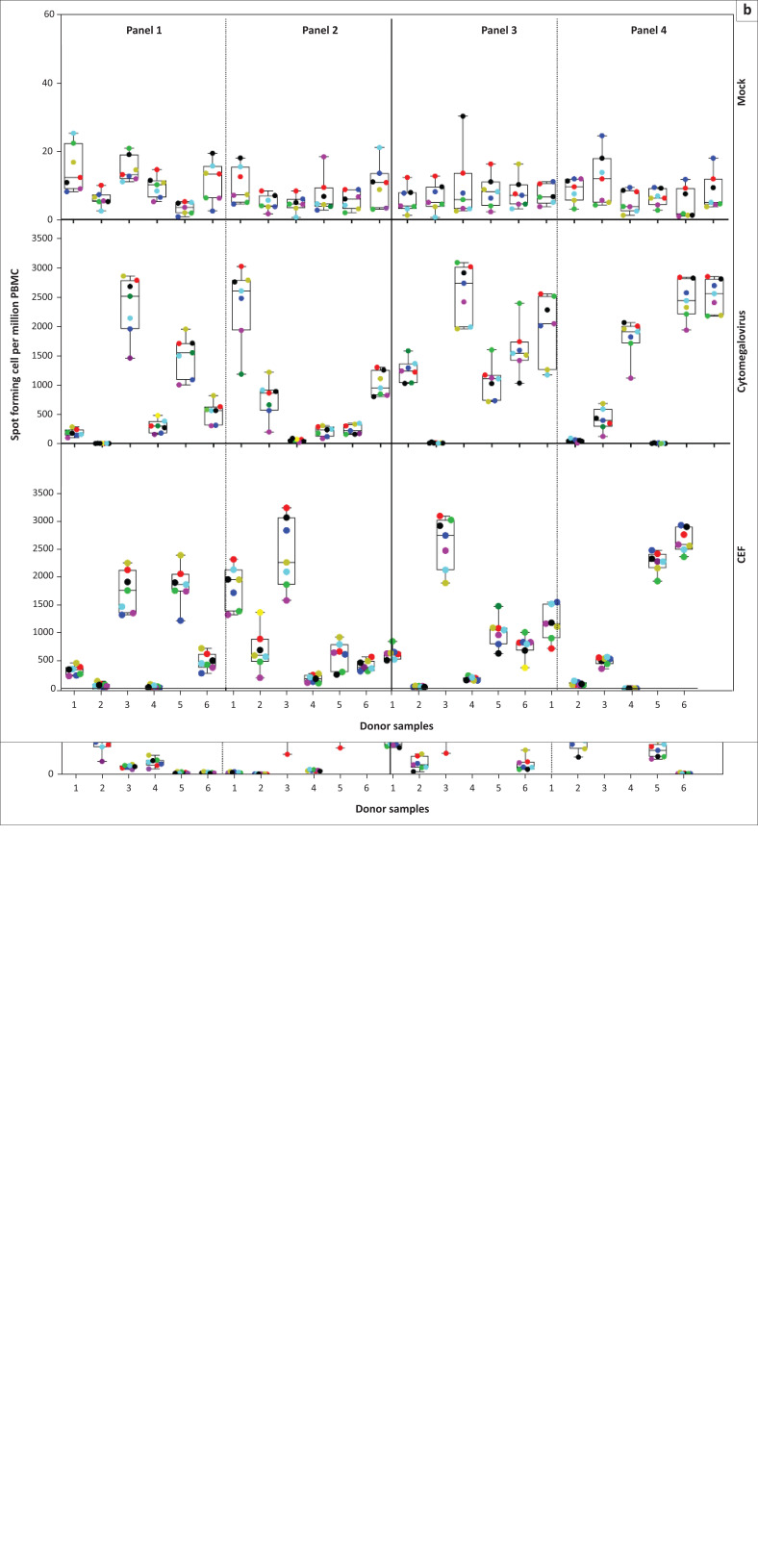

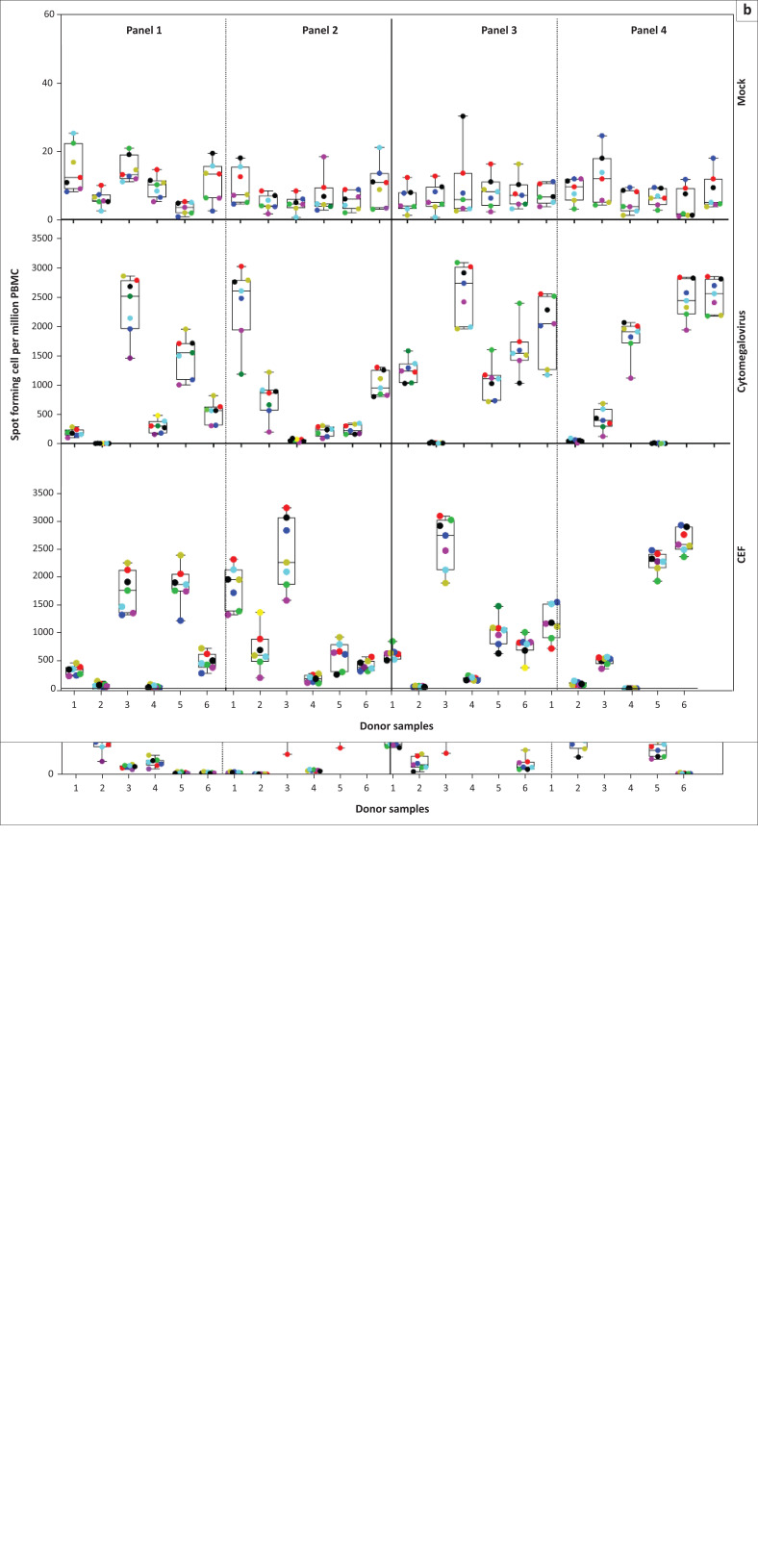
Distribution of ELISpot responses across laboratories in Kenya, Uganda, Rwanda, Zambia, South Africa and the United Kingdom, 2010–2014. Responses against mock, cytomegalovirus and cytomegalovirus, Epstein-Barr virus, and influenza virus stimuli from a panel of six peripheral blood mononuclear cells tested over 6 months, (a) first 24 months and (b) second 24 months. Box plots represent the quartiles, horizontal line the median and whiskers the maximum and minimum values. Each point represents average spot-forming cells per 10^6^ peripheral blood mononuclear cells from replicates per donor at each laboratory. The laboratories are color-coded as follows: Kenya AIDS Vaccine Initiative-Institute of Clinical Research (red); Uganda Virus Research Institute (blue); Projet San Francisco (green); Zambia EMORY HIV Research Project (purple); Kenya Medical Research Institute Centre for Geographical Medicine Research Coast (yellow); Clinical Laboratory Services (cyan); Human Immunology Laboratory (black).

For each sample, four replicate ELISpot plate wells per peptide pool were assayed and the arithmetic mean was used for analysis. Results based on fewer than 4 replicate counts were assumed to be less accurate and excluded from the analysis. A total of 1751 assays were performed of which 50 were excluded (i.e. about 2.9%). For peptide pool repeated measures, the Poisson regression model was fit to background-subtracted (except mock) ELISpot counts, with counts from the same volunteer assumed to be correlated. The resulting least-squares parameter estimates are presented together with their 95% confidence intervals adjusted for multiple comparisons using the Bonferroni method. Each model included volunteer, laboratory and month as covariates. Pair-wise comparisons between laboratories are shown as the ratio of the least-squares estimates of the mean count with corresponding adjusted (Bonferroni) 95% confidence intervals. Statistical significance was defined as a 95% confidence interval for the ratio that excludes unity (i.e. entirely above or below the value 1). Figures 1, 4 and 5, and Supplementary Document 1 Figure 1 were generated by Graph Pad Prism software version 8.3.0 (GraphPad Software, San Diego, California, United States), while the rest of the figures and statistical analyses were performed using SAS Version 9.3 (SAS Institute, Cary, North Carolina, United States).

## Results

### ELISpot testing performance across seven laboratories

Almost all (1733/1751; 99%) of the ELISpot proficiency assays performed had data within acceptable ranges with low responses to mock stimuli within the acceptance criteria of less than 50 SFC per million cells across the seven laboratories over time (Figure 1). A small fraction of these (18/1751; 0.01%) PBMCs had mock responses over 50 SFC per million cells; however, on average replicates per donor, all mock responses were below 50 SFC per million cells in all the panels. All the five laboratories actively conducting immunological analyses in support of IAVI-sponsored clinical trials performed similarly in ELISpot testing with comparable data in ELISpot counts against cytomegalovirus and CEF peptides (Figure 1). The ELISpot counts were background-subtracted (except mock). The covariates in the model were sample, laboratory and month. Across the seven laboratories, the geometric mean ELISpot counts (SFC per 10^6^ PBMC) for mock stimuli were 6–10 (Supplementary Document 1 Table 1), 289–438 for CEF stimuli (Supplementary Document 1 Table 2) and 172–266 for cytomegalovirus (Supplementary Document 1 Table 3). We observed significant differences in mock counts across the laboratories with CLS mock counts estimated to be 1.73 times higher than ZEHRP and at PSF which were 0.78 times lower than UVRI. Zambia EMORY HIV Research Project tends to have lower mock counts than all other laboratories (Supplementary Document 1 Table 1; *p* < 0.001).

**FIGURE 2:**
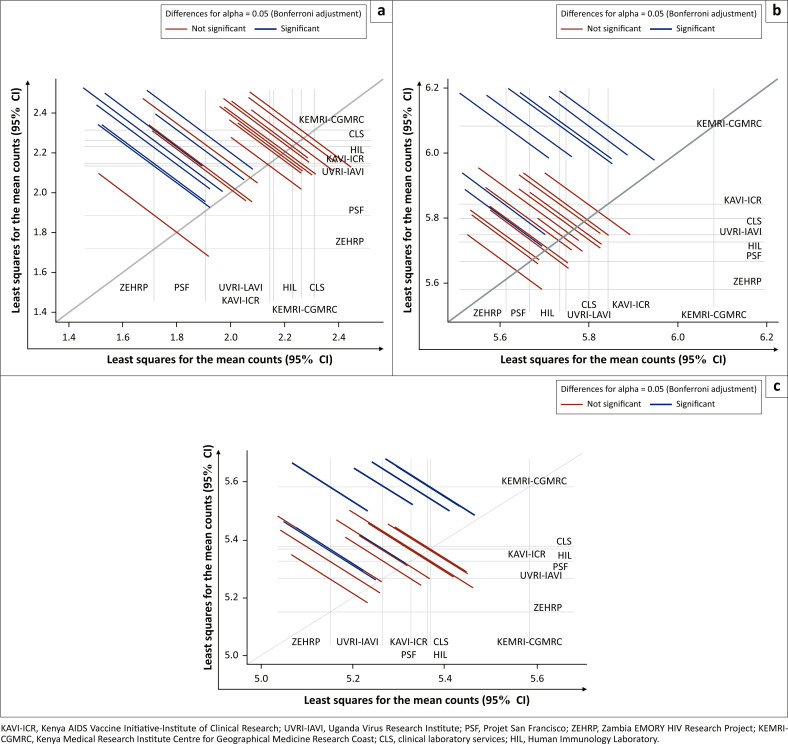
Comparison of ELISpot responses across laboratories in Kenya, Uganda, Rwanda, Zambia, South Africa and the United Kingdom, 2010–2014. The graphs show which site pairs are significantly different (blue lines) and which are not (red lines). For each comparison, a line segment, centred at the least-squares-means in the pair, is drawn. The segment length corresponds to the projected width of a confidence interval for the least-squares mean difference. Each line corresponds to the pair of labs with reference lines that cross at the midpoint. Shown here are the pair-wise least-squares means and their statistical significance, on a natural log scale, for mock, cytomegalovirus, Epstein-Barr virus, and influenza virus and cytomegalovirus stimuli. Differences for alpha = 0.05 (Bonferroni Adjustment); Red line denotes not significant while blue line denotes significant. (a) Mock, (b) cytomegalovirus, Epstein-Barr virus, and influenza virus and (c) Cytomegalovirus.

When we compared the ELISpot responses against CEF peptide pools across laboratories, KEMRI-CGMRC had significantly higher counts than other laboratories (*p* = 0.004, Figure 2 and Supplementary Document 1 Table 2). When data from KEMRI-CGMRC are excluded, the overall difference between laboratories is not statistically significant (*p* = 0.11, Supplementary Document 1 Table 2). The same trend was seen in ELISpot responses against the cytomegalovirus stimulus where KEMRI-CGMRC again had significantly higher counts than other laboratories (*p* = 0.012, Figure 2 and Supplementary Document 1 Table 3). Even after excluding data from KEMRI-CGMRC, the overall difference between laboratories is still statistically significant because of lower counts in ZEHRP than in CLS and KAVI-ICR (*p* = 0.033, Supplementary Document 1 Table 3).

### Inter-operator analysis

**FIGURE 3:**
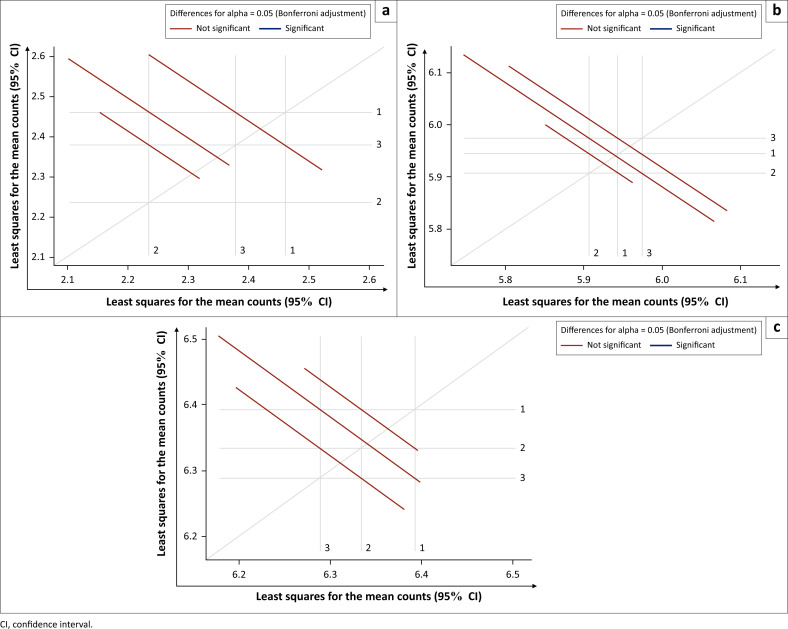
Comparison of inter-operator ELISpot responses from three operators at Kenya AIDS Vaccine Initiative-Institute of Clinical Research, Kenya, 2010–2014. The graphs show which operators are significantly different (blue lines) and which are not (red lines). Shown here are the pair-wise least-squares means and their statistical significance, on a natural log scale, for mock, cytomegalovirus, Epstein-Barr virus, and influenza virus and cytomegalovirus stimuli. For each comparison, a line segment, centred at the least-squares means in the pair, is drawn. The length of the segment corresponds to the projected width of a confidence interval for the least-squares mean difference. Segments that fail to cross the 45° reference line correspond to significant least-squares mean differences. None of the pairs of operators is significantly different (all lines cross the 45° reference line). Differences for alpha = 0.05 (Bonferroni Adjustment); Red line denotes not significant while blue line denotes significant. (a) Mock, (b) cytomegalovirus, Epstein-Barr virus, and influenza virus and (c) Cytomegalovirus.

The performance of three operators from KAVI-ICR in ELISpot testing during the study period was analysed. PBMCs from 12 volunteers were analysed by the three operators on a rotational basis with each operator conducting the same set of samples at monthly time points. ELISpot counts were obtained for mock and background-subtracted cytomegalovirus and CEF responses and the data analysed using the repeated measures Poisson regression model and comparison-adjusted against the sample, operator and month. The geometric mean ELISpot counts for mock were 9–12, 368–393 for CEF and 538–598 for cytomegalovirus stimuli (Supplementary Document 1 Table 4). We found no significant difference in ELISpot performance between the three operators (Figure 3 and Supplementary Document 1 Table 4).

### Performance of four laboratories in PBMC processing

**FIGURE 4:**
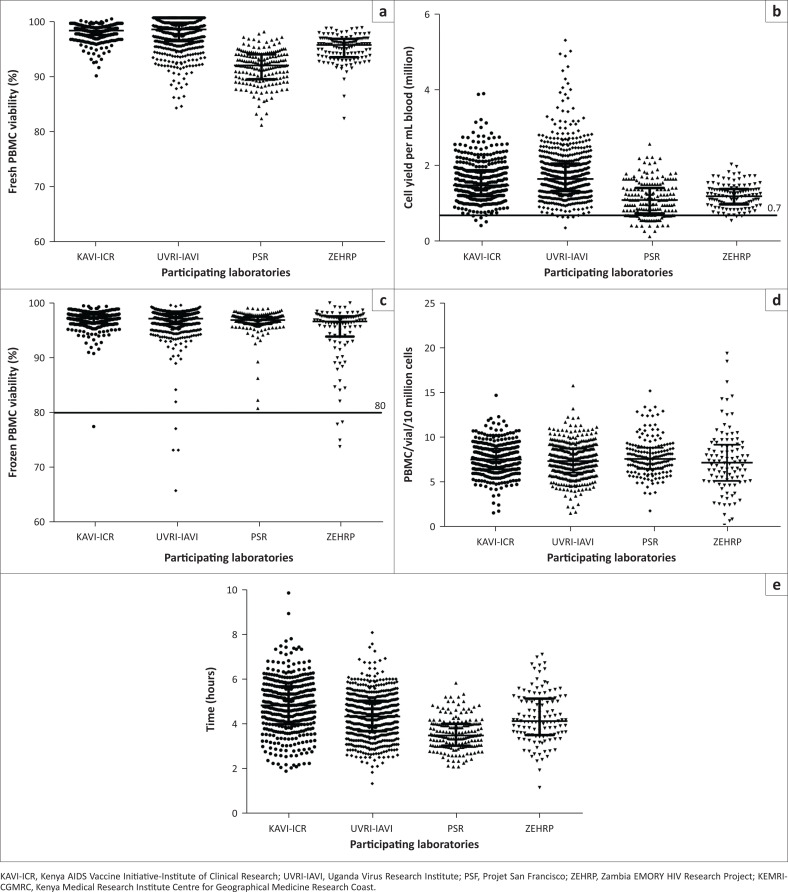
Performance of laboratories in peripheral blood mononuclear cell (PBMC) processing, Kenya, Uganda, Rwanda, Zambia, 2010–2014. (a) The percentage cell viability of freshly isolated PBMC, (b) cell yield per mL of blood, (c) percentage cell viability from frozen PBMCs, (d) cell recovery of frozen PBMC (PBMCs were cryopreserved at a final concentration of 10–15 m cells per mL; here data were normalised to 10 m cells), and (e) duration of PBMC processing (in hours). Each dot represents a sample and the horizontal line represents the median with interquartile range. The long horizontal line shows the acceptance cut-off.

A total of 1297 PBMCs isolated from clinical trial samples at the four aforementioned laboratories supporting two IAVI-sponsored HIV clinical trials were analysed for cell viability, recovery and cell yield per mL of blood. Of the 1297 PBMCs processed, 1220 (94%) freshly isolated PBMCs had viability above 90% with a median of 95% (range 81% – 100%) and those with viability below 90% had a median of 88% (range 81% – 90%) (Figure 4a). Over 96% (1249/1297) of these samples had cell yields greater than 0.7 × 10^6^ PBMC per mL blood, all within the predefined acceptability criteria (Figure 4b). There were a few samples that had low cell yield ranging from 0.13 to 0.56 × 10^6^ PBMC per mL blood (Figure 4b). A fraction of these samples (1205/1297; 93%) including those with cell yield below 0.7 × 10^6^ PBMC per mL blood were thawed and tested for ELISpot performance and almost all (1196/1205; 99%) PBMCs had viability above 80% following thaw and overnight rest (within acceptability criteria) and only 9 PBMCs were below 80% (range 66% – 78%) (Figure 4c). The cell recovery for these samples was above 6.0 × 10^6^ PBMC per vial (data were normalised to 10 m cells (Figure 4d); samples from the clinical trials analysed in this study were frozen at 10 m cells per mL except one trial where samples were frozen at 15 m cells per mL per vial at which the data were normalised to 10 m cells per mL for uniformity of data. In ELISpot testing, all the samples were functional with over 95% having mock responses under 50 SFC per 10^6^ PBMC, PHA over 1000 SFC per 10^6^ PBMC and a range of cytomegalovirus responses.

The duration of PBMC processing from blood draw to cryopreservation has been shown to affect the integrity of cells.^23,24,25^ In this study, we analysed the processing time of PBMC (in hours) and report that nearly all the samples were processed within 6 h with only 6% (81/1297) processed beyond 6 h (range 6.1–9.5 h) (Figure 4e and Supplementary Document 1 Figure 1). To assess the integrity of samples processed past 6 h, we analysed the PBMC viability and cell yield from freshly isolated PBMCs and later analysed the viability and cell recovery from thawed frozen PBMCs. We found the delays did not affect the PBMCs’ integrity in all samples; one had cell viability above 90% and a cell yield greater than 0.7 × 10^6^ PBMC per mL blood (both parameters within acceptable range) (Supplementary Document 1 Figure 1). Only one sample had a slightly lower cell yield of 0.57 × 10^6^ per mL blood and cell viability of 98%. Post PBMC freezing cell viability ranged from 93% to 100% and cell recovery was above 6 × 10^6^ PBMC per vial in 71 of 81 (87%) samples (Supplementary Document 1 Figure 1). We further tested these samples in an ELISpot assay to assess their cell functionality and all samples performed well with the mock responses under 50 SFC per 10^6^ PBMC, PHA responses greater than 1000 SFC per 10^6^ PBMC and a typical range of cytomegalovirus responses. These data are similar to what was seen from samples processed within 6 h (Supplementary Document 1 Figure 1).

## Discussion

We compared data generated in multiple laboratories over time to assess their performance in the processing of PBMC samples and ELISpot testing. Some of these laboratories supported clinical trials of HIV prophylactic vaccine candidates and to standardise their cell functionality assays and harmonise procedures to achieve reliable and accurate data, they were enrolled in an ELISpot proficiency scheme as part of external quality assurance. All laboratories performed well in ELISpot proficiency testing with data comparable across laboratories; however, there were a few sporadic outliers in the data. These outliers were expected considering the large number of data points analysed: sporadic outliers would be expected even from experienced and competent laboratories. In this study, we saw comparable ELISpot data from five out of seven laboratories. These five laboratories all supported clinical trials except one: CLS which performed ELISpot regularly. Since CLS was the laboratory responsible for sourcing and qualifying the PBMCs for the ELISpot proficiency scheme, they were expected to perform well in ELISpot testing. In the two laboratories that did not support clinical trials and routine ELISpot testing, we saw significant differences in ELISpot proficiency data compared to other laboratories. It is worth mentioning that these laboratories performed ELISpot testing quarterly and would be less experienced compared to other laboratories. To identify the root cause of this data discrepancy, corrective action was initiated at these two laboratories. The staff were retrained and assessed for competency. Also, improvement measures were put in place such as continuous training and monitoring of their performance in ELISpot testing in subsequent rounds of proficiency testing panels.

As a requirement of the GCLP programme, at any given time, there should be more than one person processing the clinical trial samples to ensure accuracy and reliability of data. Likewise, in ELISpot proficiency testing, different operators at each laboratory fully trained in the required procedures conducted ELISpot proficiency assays on a rotational basis as determined by laboratory management. The influence of the operator on the variability of results is a known factor as shown by Janetzki and colleagues;^14^ here we analysed the performance of three operators in ELISpot proficiency testing in one laboratory which maintained the same staff throughout the study period. It was not possible to analyse inter-operator variability in all laboratories as many sites experienced frequent staff turnover during the study period. From the analysis, there was no significant difference in data generated by the three operators over the study period. This demonstrates that with regular training and competency assessment, reliable, accurate and comparable data can be obtained notwithstanding the inherent operator variability.

Sample integrity is critical for achieving accurate and reliable results in clinical trials. In a multicentre trial, processes for sample processing need to be harmonised to generate comparable data. We assessed the processing of PBMCs in four of seven laboratories, focusing on five areas: sample collection, isolation of PBMC, cryopreservation, thawing of frozen PBMC, and performance in ELISpot testing of clinical trial samples. First, all laboratories were required to process samples to cryopreservation within 6 h of a blood draw. This is to ensure that PBMCs obtained are of good quality as it has been documented by Olson and colleagues that a delay in the processing of PBMC of more than 8 h may reduce cell viability and compromise cell functionality.^26^ We found most samples were processed within 6 h with few samples outside of this time frame. The few samples were processed outside this time frame due to reasons such as a delay in delivery to the laboratory, as some clinics were some distance from the laboratory, batching of sample processing and a backlog of samples. To mitigate these, corrective and preventative actions were initiated to prevent such recurrence which included prioritising the delivery of samples from the clinics, processing samples promptly as they arrive in the laboratory and cryopreserving PBMC immediately after processing to reduce any backlog that may have arisen from batching of cryopreservation. These measures initiated were monitored monthly. Although some samples were processed beyond the expected time frame, their integrity was not compromised as cell viability, cell yield and recovery after thawing were indistinguishable from other samples processed within the stipulated time. Additionally, these samples were tested for functionality in downstream assays such as ELISpot and they performed well, again with indistinguishable results compared with samples processed within the stipulated time. In conclusion, we have shown that samples processed beyond 6 h and up to 9 h from blood draw to cryopreservation are still viable and functional, similar to findings from other groups.^24,25^

In assessments of PBMC isolation, our focus was on cell viability and cell yield. We found that the majority of PBMC samples isolated in all four laboratories supporting clinical trials met the predefined acceptance criteria for viability and cell yield with few outliers seen across the laboratories.

In most cases, samples obtained in a multicentre clinical trial are shipped to a central laboratory either for long-term storage or cellular functionality testing. Therefore, proper cryopreservation after PBMC isolation and shipping is critical in preserving cell integrity and functionality.^27^ Clinical trial samples isolated at four laboratories were cryopreserved at the local freezing repository until shipped to a central laboratory. Upon request, the samples were shipped to the central laboratory according to the IATA guidelines for long-term storage. The majority of thawed samples were viable with cell viability and recovery within the acceptable limits and thus demonstrated the competency of the laboratories in cryopreservation and shipping of samples. Additionally, thawed samples performed well in ELISpot testing with responses within the expected ranges.

To maintain high standards and produce reliable and comparable data, these laboratories were audited regularly for GCLP compliance either by internal or external independent auditors. The audit covers areas such as operating procedure development and documentation, ELISpot and flow cytometry testing proficiency schemes and the data management system. All laboratories were audited annually by an external auditor for GCLP accreditation.

### Limitations

The main limitation of this study was the variation in the operators who performed ELISpot testing at the laboratories. There was a frequent turnover of personnel in most of the laboratories during the study period which made it difficult to assess the inter-operator variability within sites. To assess the operator effect on data variability, data were analysed from only one laboratory which had three operators who consistently performed ELISpot testing over the 4 years on a rotational basis. Though we saw no significant difference in data generated between these three operators, this result does not entirely represent what could be seen across all seven laboratories.

### Conclusion

In conclusion, we have demonstrated the capabilities of multiple laboratories in Africa and Europe in processing clinical trial samples to high standards and performing cell functionality assays. Furthermore, we have shown that multiple laboratories can generate reliable, accurate and comparable data by using standardised procedures, having regular training, regular equipment maintenance and using centrally sourced reagents. These efforts and the ELISpot proficiency programme continue across the network of IAVI-sponsored laboratories supporting clinical trials. Therefore, we highly recommend the approach taken by the IAVI GCLP-accredited laboratories to produce such data to any donor, sponsor or research institution who may plan to conduct clinical trials in the region.

## References

[1] Gilmour JW, Stevens WS, Gray C, De Souza M. Laboratory expansion to large-scale international HIV preventive vaccine trials. Curr Opin HIV AIDS. 2007;2(3):201–206. 10.1097/COH.0b013e3280eec77a 19372887

[2] Kublin JG, Morgan CA, Day TA, et al. HIV vaccine trials network: Activities and achievements of the first decade and beyond. Clin Investig. 2012;2(3):245–254. 10.4155/cli.12.8 PMC3521567 23243491

[3] Muyanja E, Ssemaganda A, Ngauv P, et al. Immune activation alters cellular and humoral responses to yellow fever 17D vaccine. J Clin Investig. 2014;124(7):3147–3158. 10.1172/JCI75429 24911151 PMC4071376

[4] Schmidt C, Jaoko W, Omosa-Manyonyi G, et al. Long-term follow-up of study participants from prophylactic HIV vaccine clinical trials in Africa. Hum Vaccines Immunother. 2014;10(3):714–723. 10.4161/hv.27559 PMC4130282 24374365

[5] Mwangoka G, Ogutu B, Msambichaka B, et al. Experience and challenges from clinical trials with malaria vaccines in Africa. Malar J. 2013;12(1):86. 10.1186/1475-2875-12-86 23496910 PMC3599886

[6] Kent DM, Mwamburi DM, Bennish ML, Kupelnick B, Ioannidis JP. Clinical trials in sub-Saharan Africa and established standards of care: A systematic review of HIV, tuberculosis, and malaria trials. JAMA. 2004;292(2):237–242. 15249573 10.1001/jama.292.2.237

[7] Stiles T, Grant V. Good Clinical Laboratory Practice (GCLP): An international quality system for laboratories which undertake the analysis of samples from clinical trials. Ipswich: Research Quality Association (RQA); 2012.

[8] Sarzotti-Kelsoe M, Cox J, Cleland N, et al. Evaluation and recommendations on good clinical laboratory practice guidelines for phase I–III clinical trials. PLoS Med. 2009;6(5):e1000067. 10.1371/journal.pmed.1000067 19536325 PMC2670502

[9] Baden LR, Karita E, Mutua G, et al. Assessment of the safety and immunogenicity of 2 novel vaccine platforms for HIV-1 prevention: A randomized trial. Ann Intern Med. 2016;164(5):313–322. 26833336 10.7326/M15-0880 PMC5034222

[10] Omosa-Manyonyi G, Mpendo J, Ruzagira E, et al. A Phase I double blind, Placebo-controlled, randomized study of the safety and immunogenicity of an adjuvanted HIV-1 Gag-Pol-Nef fusion protein and adenovirus 35 Gag-RT-Int-Nef vaccine in healthy HIV-uninfected African adults. PLoS One. 2015;10(5):e0125954. 10.1371/journal.pone.0125954 25961283 PMC4427332

[11] Mpendo J, Mutua G, Nyombayire J, et al. A Phase I double blind, Placebo-controlled, randomized study of the safety and immunogenicity of electroporated HIV DNA with or without interleukin 12 in prime-boost combinations with an Ad35 HIV vaccine in healthy HIV-seronegative African Adults. PLoS One. 2015;10(8):e0134287. 10.1371/journal.pone.0134287 26252526 PMC4529153

[12] Hanke T, Mutua G, Farah B, et al. Broad HIV-1 inhibition in vitro by vaccine-elicited CD8(+) T cells in African adults. Mol Ther Methods Clin Dev. 2016;3:16061. 10.1038/mtm.2016.61 27617268 PMC5006719

[13] Klausner RD, Fauci AS, Corey L, et al. Medicine. The need for a global HIV vaccine enterprise. Science (New York, NY). 2003;300(5628):2036–2039. 10.1126/science.1086916 12829768

[14] Janetzki S, Schaed S, Blachere NE, Ben-Porat L, Houghton AN, Panageas KS. Evaluation of Elispot assays: Influence of method and operator on variability of results. J Immunol Methods. 2004;291(1–2):175–183. 10.1016/j.jim.2004.06.008 15345315

[15] Cox JH, Ferrari G, Kalams SA, Lopaczynski W, Oden N, D’Souza MP. Results of an ELISPOT proficiency panel conducted in 11 laboratories participating in international human immunodeficiency virus type 1 vaccine trials. AIDS Res Hum Retroviruses. 2005;21(1):68–81. 10.1089/aid.2005.21.68 15665646

[16] Sanchez AM, Rountree W, Berrong M, et al. The external quality assurance oversight laboratory (EQAPOL) proficiency program for IFN-gamma enzyme-linked immunospot (IFN-γ ELISpot) assay. J Immunol Methods. 2014;409:31–43. 10.1016/j.jim.2014.03.017 24685833 PMC4138255

[17] Gill DK, Huang Y, Levine GL, et al. Equivalence of ELISpot assays demonstrated between major HIV network laboratories. PLoS One. 2010;5(12):e14330. 10.1371/journal.pone.0014330 21179404 PMC3001861

[18] Boaz MJ, Hayes P, Tarragona T, et al. Concordant proficiency in measurement of T-cell immunity in human immunodeficiency virus vaccine clinical trials by peripheral blood mononuclear cell and enzyme-linked immunospot assays in laboratories from three continents. Clin Vaccine Immunol. 2009;16(2):147–155. 10.1128/CVI.00326-08 19091991 PMC2643552

[19] Rountree W, Vandergrift N, Bainbridge J, Sanchez AM, Denny TN. Statistical methods for the assessment of EQAPOL proficiency testing: ELISpot, Luminex, and Flow Cytometry. J Immunol Methods. 2014;409:72–81. 10.1016/j.jim.2014.01.007 24456626 PMC4104253

[20] Liese BH, Houghton N, Teplitskaya L. Development assistance for neglected tropical diseases: Progress since 2009. Int Health. 2014;6(3):162–171. 10.1093/inthealth/ihu052 25096331

[21] Currier JR, Kuta EG, Turk E, et al. A panel of MHC class I restricted viral peptides for use as a quality control for vaccine trial ELISPOT assays. J Immunol Methods. 2002;260(1–2):157–172. 10.1016/S0022-1759(01)00535-X 11792386

[22] Britten CM, Janetzki S, Butterfield LH, et al. T cell assays and MIATA: The essential minimum for maximum impact. Immunity. 2012;37(1):1–2. 10.1016/j.immuni.2012.07.010 22840835

[23] Mallone R, Mannering SI, Brooks-Worrell BM, et al. Isolation and preservation of peripheral blood mononuclear cells for analysis of islet antigen-reactive T cell responses: Position statement of the T-Cell Workshop Committee of the Immunology of Diabetes Society. Clin Exp Immunol. 2011;163(1):33–49. 10.1111/j.1365-2249.2010.04272.x 20939860 PMC3010910

[24] Kierstead LS, Dubey S, Meyer B, et al. Enhanced rates and magnitude of immune responses detected against an HIV vaccine: Effect of using an optimized process for isolating PBMC. AIDS Res Hum Retroviruses. 2007;23(1):86–92. 10.1089/aid.2006.0129 17263637

[25] Bull M, Lee D, Stucky J, et al. Defining blood processing parameters for optimal detection of cryopreserved antigen-specific responses for HIV vaccine trials. J Immunol Methods. 2007;322(1–2):57–69. 10.1016/j.jim.2007.02.003 17382342 PMC1904432

[26] Olson WC, Smolkin ME, Farris EM, et al. Shipping blood to a central laboratory in multicenter clinical trials: Effect of ambient temperature on specimen temperature, and effects of temperature on mononuclear cell yield, viability and immunologic function. J Transl Med. 2011;9:26. 10.1186/1479-5876-9-26 21385453 PMC3063218

[27] Smith JG, Joseph HR, Green T, et al. Establishing acceptance criteria for cell-mediated-immunity assays using Frozen peripheral blood mononuclear cells stored under optimal and suboptimal conditions. Clin Vaccine Immunol. 2007;14(5):527–537. 10.1128/CVI.00435-06 17376862 PMC1865640

